# Modelling the resilience of rail passenger transport networks affected by large-scale disruptive events: the case of HSR (high speed rail)

**DOI:** 10.1007/s11116-018-9875-6

**Published:** 2018-04-18

**Authors:** Milan Janić

**Affiliations:** 1 Transport and Planning Department, Faculty of Civil Engineering and Geosciences, Delft University of Technology, Stevinweg 1, 12628 BX Delft, The Netherlands; 2 Air Transport and Operations Department, Faculty of Aerospace Engineering, Delft University of Technology, Stevinweg 1, 12628 BX Delft, The Netherlands

**Keywords:** Rail passenger transport networks, Performances, Indicators, Resilience, HSR (high speed rail) case

## Abstract

This paper deals with modelling the dynamic resilience of rail passenger transport networks affected by large-scale disruptive events whose impacts deteriorate the networks’ planned infrastructural, operational, economic, and social-economic performances represented by the selected indicators. The indicators of infrastructural performances refer to the physical and operational conditions of the networks’ lines and stations, and supportive facilities and equipment. Those of the operational performances include transport services scheduled along particular routes, their seating capacity, and corresponding transport work/capacity. The indicators of economic performances include the costs of cancelled and long-delayed transport services imposed on the main actors/stakeholder involved—the rail operator(s) and users/passengers. The indicators of social-economic performances reflect the compromised accessibility and consequent prevention of the user/passenger trips and their contribution to the local/regional/national Gross Domestic Product. Modeling resulted in developing a methodology including two sets of analytical models for: (1) assessing the dynamic resilience of a given rail network, i.e., before, during, and after the impacts of disruptive event(s); and (2) estimation of the indicators of particular performances as the figures-of-merit for assessing the network’s resilience under the given conditions. As such, the methodology could be used for estimating the resilience of different topologies of rail passenger networks affected by past, current, and future disruptive events, the latest according to the “what-if” scenario approach and after introducing the appropriate assumptions. The methodology has been applied to a past case—the Japanese Shinkansen HSR network affected by a large-scale disruptive event—the Great East Japan Earthquake on 11 March 2011.

## Introduction

Resilience has been defined differently, mainly depending on the systems considered. In general, for engineering systems, it has been defined as the sum of the passive survival rate (reliability) and the proactive survival rate (restoration) (Youn et al. 2011). Resilience has also been considered as an intrinsic ability of systems to adjust their functionality in the presence of disturbances and unpredicted changes (Hollnagel et al. 2006). Furthermore, it has been regarded as the ability of the systems to sustain the impacts of external and internal disruptions without discontinuity of performing their function; or if these functions are disconnected, to recover them rapidly and completely (ASME 2009). In particular, the resilience of transport systems as sub-components of engineering systems has been defined as their ability to predict, absorb, adapt, and/or quickly recover after the impact of disruptive events such as, for example, natural disasters (NIAC 2009). Since the transport sector and its infrastructure have been recognized as important contributors to the national economies and societies, most research in the given context has dealt with their resilience (Percoco 2004).

The above-mentioned concepts and definitions of resilience can also be applied, after the necessary modifications, to rail passenger networks—both conventional and HSR (high speed rail)^1^ as components of the transport network/system of many countries worldwide. Dealing with the resilience of these networks usually implies considering how their selected planned performances change if affected by the impact of various internal and external disruptive events. Most often, the consequences depending on the intensity and duration of these impacts include physical damage to infrastructure, failures of components of the supporting facilities and equipment and rolling stock, and consequently cancellations and/or relatively long delays of the affected transport services (Ip and Wang 2011). The main directly affected actors/stakeholders have always been on the supply side the rail transport service operator(s), i.e., providers of transport infrastructure and services, and on the demand side the system’s users/passengers. In some cases, the dependent businesses and governments at different institutional scales—local/regional/national—are also affected. In particular, the rail operator(s) and its users/passengers can generally be imposed the direct costs associated with the affected performances during and after the impact of disruptive events, i.e., during their deteriorating and recovering, respectively. This has raised the question of the resilience of the existing conventional and particularly HSR passenger networks affected by different disruptive events, particularly large-scale ones.

Therefore, this paper aims to addressing precisely this question. In addition to this introductory section, the paper consists of four other sections. “Some characteristics of rail passenger networks and the concept of their resilience” Section describes the relevant characteristics of conventional and HSR network(s) and the concept of their resilience. “Modeling resilience of the affected rail passenger networks” Section presents the methodology consisting of the analytical models for estimating resilience of these networks and the models of indicators of selected performances as the figures-of-merit for assessing it. “An application of the methodology to the affected HSR (high speed rail) network” Section presents an application of the proposed methodology ex post, i.e., to a past case, namely to the Japanese Shinkansen HSR network, which was affected by a large-scale disruptive event, the Great East Japan Earthquake on 11 March 2011. The last section summarizes some conclusions.

## Some characteristics of rail passenger networks and the concept of their resilience

### Components, spatial configuration, and operations

**Fig. 1:**
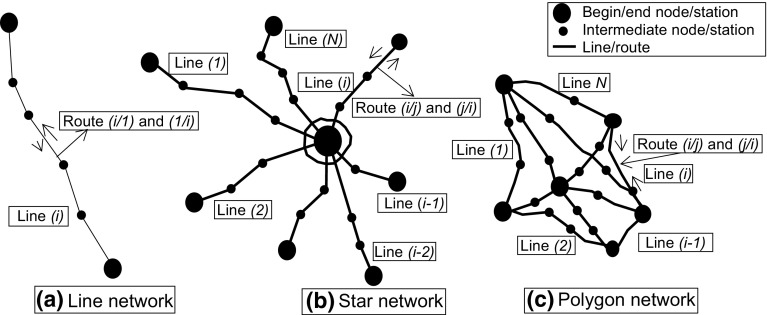
Simplified topology of the HSR (high speed rail) network(s).(Reproduced with permission from Crozet 2013; http://www.johomaps.com/eu/europehighspeed.html/)

In general, rail passenger networks (either conventional or HSR) consist of fixed and mobile physical components (excluding the operating staff). The fixed components include the networks’ infrastructure—stations as nodes, and rail lines with tracks as links connecting these nodes, the supportive facilities and equipment (traffic control/signaling, power supply, and traffic management system), and the maintenance systems of infrastructure and rolling stock. The mobile components include the rolling stock—trains—carrying out the transport services. In different countries, the railway infrastructure, both conventional and HSR, has been generally built line-by-line including the intermediate and end stations, thus creating the infrastructure networks usually spreading between the main urban agglomerations. Such development has made the topology, i.e., spatial layout/configuration, of these networks mainly the country specific. Figure 1 shows three typical topologies of the HSR networks.

For example, topology (a)-Line network reflects the layout of real HSR networks in Italy and Japan; topology (b)-Star network reflects the layout of real HSR networks in France and Spain; and topology (c)-Polygon network reflects the layout of the real HSR network in Germany (Crozet 2013; Janić 2016; http://www.johomaps.com/eu/europehighspeed.html/).

Conventional or HS (High Speed) trains are scheduled along particular lines to serve the user/passenger flows. These can have different O-Ds (Origins and Destinations) at the terminuses/stations along the lines thus defining the routes along them.

### Disruptive events, their impacts, and related costs

The above-mentioned topologies of either conventional or HSR networks resist the impacts of particular disruptive events differently. For example, if a disruptive event affects a certain station/node of a Line network, the transport services between the stations/nodes on both sides of location of the impact will be affected, i.e., usually cancelled or long-delayed. In a Star network, the impact of a disruptive event can affect transport services throughout the entire network if, for example, it takes place at the central node—station. In addition, the transport services on particular lines/routes can also be affected, i.e., cancelled or long-delayed, similarly as in a Line network. In a Polygon network, the particular lines/routes can be similarly affected as in a Line network, but the remaining ones will usually stay in the planned state, i.e., fully operational.

Generally, disruptive events affecting rail passenger networks can be external and internal.

- *Internal disruptive events* can be severe failures of the main network components such as rolling stock, supporting facilities and equipment, and/or infrastructure (rails) compromising or preventing safe operation of trains, and industrial actions of the railway staff. These events deteriorate the planned performances of a given rail network and/or its components, thus usually causing long delays and/or cancellations of the affected transport services. Sometimes, some of these internal events can cause fatal incidents/accidents resulting in user/passenger and crew injuries and/or fatalities, and damages to the close surroundings as well (NDTnet 2000; Qiao 2012; Puente 2014).
- *External disruptive events* can be severe weather such as: heavy rain with flooding [for example, in Europe Saxony (2002), Alpine (2005), UK (2007 and 2012), and Central Europe (2013)]; severe winds/storms (for example, in Europe the storms Lothar and Martin (1999), Gudrun (2005), and Kyrill (2007), and in the U.S. Hurricane Sandy (2012)); and heavy snow/winter conditions [for example, in Europe in Sweden (2001–2002), and Western Europe (2009–2010)] (Jaroszweski et al. 2014). In particular, the above-mentioned snowfalls mainly affect the individual transport services of both conventional and HSR causing their blockage for a certain period of time.^2^ In addition, they sometimes cause incidents/accidents such as derailment of the affected HS trains (https://en.wikipedia.org/wiki/List_of_TGV_accidents).
- *Other external disruptive events* include natural disasters such as earthquakes. An illustrative example as the most severe case further elaborated in this paper, was the Great East Japan Earthquake, which occurred on 11 March 2011, whose impact severely affected the Tohoku Shinkansen HSR line and consequently the entire Shinkansen network (Kazama and Noda 2012; Nakamura 2011; Shimamura and Keyaki 2013).
- *Specific external disruptive events* include terrorist threats and attacks. For example, one such attack on a HSR network—bombing of transport service on the Marseille–Paris route on 31 December 1983—caused five fatalities and 50 injuries. Another was the terrorist attack in France on board a Thalys HS train on its way from Amsterdam to Paris on 21 August 2015 (http://www.trainweb.org/tgvpages/tgvindex.html/; https://en.wikipedia.org/wiki/List_of_terrorist_incidents_in_France; https://en.wikipedia.org/wiki/2015_Thalys_train_attack). In addition, the most recent terrorist attacks on the airport and metro station in Brussels (Belgium) on 22 March 2016, although not directly impacting, caused the cancellation of all Eurostar and Thalys HSR services to and from the Brussels Midi station over the entire day (http://www.bloomberg.com/news/articles/2016-03-22/air-train-travel-slows-in-europe-after-brussels-airport-bombing).

The impacts of the above-mentioned disruptive events, generally occurring randomly (and unpredictably) in time and space/location, can directly affect the planned performances of affected rail networks on different spatial scales, from the local node-station/route-link-line/transport service to the global network (several nodes-stations/routes-links-lines/transport service) level. In some cases, independently of time, the spatial scale and intensity of impacts, different disruptive events may occur simultaneously, and as such their impacts can be interrelated.

In addition to deteriorating the planned infrastructural and operational performances, these impacts consequently also directly affect the corresponding economic and social-economic performances by imposing additional costs on the particular main actors/stakeholders involved—in this case these are the transport service operators, users/passengers, and consequently society. The first—providers of transport services and infrastructure—can generally be imposed the costs in terms of losses of profits from the cancelled and/or long-delayed transport services as well as the very substantial costs of repairing or even rebuilding the damaged infrastructure, and repairing the facilities and equipment and rolling stock, respectively. The second—users/passengers—can suffer from direct costs of the lost time from the long-delayed and cancelled transport services. The last—in the broader context, the society—can be imposed the costs as non-contribution or losses of the local/regional/national GDP from the non-realized user/passenger trips due to compromised accessibility. The indirect impacts of disrupted rail passenger networks on the close environment and other dependent social-economic activities and businesses have not been considered in detail.

### The concept of resilience

#### Definition and framework

The resilience of a given rail passenger network, either conventional or HSR, can be considered in different ways. Some of these are static and dynamic. The former refers to the network’s ability to maintain its specified performances during the impact of disruptive events. The later implies the network’s speed of recovery afterwards up to the state characterized by the previously planned performances. As well, in the given context, both static and dynamic resilience can be considered in the short-, medium-, and long-term, and assessed at three layers as follows (Chen and Miller-Hooks 2012; Janić 2015; Njoka and Raoult 2009; Rose 2009):

- Physical layer dealing with the physical impact of disruptive events on the network’s above-mentioned planned infrastructural performances;
- Transport service layer mainly relating to the impact of disruptive events on the network’s above-mentioned planned operational performances; and
- Cognitive layer, which as a part of the economic and social-economic performances, relates to the user/passenger renewed confidence in the network’s gradually restored infrastructural and operational performances and consequently accessibility (Leu et al. 2010).

#### Some resilience strategies for mitigating impacts of disruptive event(s)

Different resilience strategies can be applied for mitigating impacts of disruptive events on rail passenger networks (Cox et al. 2011; Rose 2009; Shimamura and Keyaki 2013). In particular, according to Cox et al. (2011) and Rose (2009), two types of strategies are generally available: those, which mitigate losses at the microeconomic scale, and those contributing to speed recovery, i.e., to dynamism of recovery of the affected system afterwards. In the given context, depending on the generic characteristics of transport services and the impact of the large-scale disruptive event(s) on the rail passenger networks, the particular resilience strategies mainly applied to the rail operator(s) can be as follows:

- ‘Conservation’ implies reducing the volume of transport services by cancelling some or all of them, and thus simultaneously constraining users’/passengers’ access to the system;
- ‘Input’ or ‘modal substitution’ can be partially applied by deploying substitutive transport services to replace the cancelled and/or long-delayed rail services. This is practiced if and where reasonable and if these substitutive transport modes and their systems have remained unaffected, i.e., operative at least at a certain scale;
- ‘Production recapture’ is applicable after repairing the damaged infrastructure, facilities and equipment, by restoring transport services safely;
- ‘Logistics refinement’ or ‘logistics delivery’ can be applied to support the ‘input’ or ‘modal substitution’ strategy by contracting transport service providers of other transport modes to take over the users/passenger flows from the cancelled and/or long-delayed rail services;
- The ‘management of effectiveness’, ‘import substitution’, ‘speeding restoration’, and ‘removing operational impediments’ strategies imply engaging material resources, spare parts, and necessary skills (also in some cases some or all of them imported from other regions) and reducing barriers to their acquiring for repairing particularly damaged infrastructure, and facilities and equipment. In addition, this strategy includes determining and deploying the order of actions on restoration of the affected rail network’s resilience and undertaking the administrative procedures to again restore operations and consequently compensate the costs to the affected stakeholders/parties; and
- The ‘risk management’ strategy, traditionally focused on reducing the likelihood of impact of disruptive events (the system’s internal strategies), potential consequences of such events by prevention (the system’s internal strategies), and protection (the system’s external strategies) area is also applicable, although in some specific cases.^3^

The other strategies mentioned by Cox et al. (2011) and Rose (2009) are not applicable in the given context due to the following reasons:

- ‘Inventories’ is inapplicable because transport services cannot be stored as ‘inventories’ and consumed later on since they are consumed at the same time as they are produced;
- ‘Excess capacity’, i.e., the lack of redundancy back-offs, is not applicable in cases of severe damage to infrastructure—rail tracks, and supportive facilities and equipment—causing large-scale cancellations of transport services. In addition, this strategy is inapplicable in cases of failures of the rolling stock when there is a lack of back-up rolling stock to be deployed;
- ‘Technological change’ is inapplicable since the existing technology always remains in place; and
- ‘Relocation’, i.e., changing the location of businesses and services (other than transport) due to the deteriorated transport services, ‘export substitution’, i.e., ‘selling’ transport services to other parts of the network, and ‘resource unimportance’, i.e., continuing a part of transport services without the critical inputs from different parties, are not considered as relevant in the given context.

## Modeling resilience of the affected rail passenger networks

### Some related research

Much research has been carried out over the past two decades and a half on the resilience of different systems. This has been primarily due to the more frequent impacts on these systems by usually unpredictable disruptive events. An exhaustive and detailed overview of the existing research has been carried out relatively recently (Hosseini et al. 2016). Out of 144 considered academic references mostly from scientific journals, 11 explicitly deal with the resilience of transport systems. In addition to analyzing domains in which the resilience and its measures have been considered, the transport resilience-related research has dealt with the analysis of definitions, qualitative and quantitative approaches to assessment of resilience. In particular, the quantitative approach has included: (1) the general measures based on deterministic and probabilistic approach, and the structural-based models including the general measures; (2) optimization models; (3) simulation models; and (4) fuzzy logic models.

- *General measures of resilience* of different systems, including transport systems and their networks actually consisting of indicators and measures (Berdica 2002; Omer et al. 2013). One of the characteristic indicators was the proportion of demand served by an intermodal freight transport network within the pre-determined recovery budget after the end of impact of a given disruptive event (Chen and Miller-Hooks 2012). A similar approach was applied by Janić (2015) to deal with the resilience of an air transport network affected by a large-scale disruptive event. In this case, the indicator of network resilience was the proportion of flights carried out on time under (given) disruptive conditions of the affected airport network. In addition, Henry and Ramirez-Marquez (2012) developed the concept and model for assessing the time-dependent resilience means by an indicator such as the ratio of recovery to losses. The system performances before, during, and after the impact of the disruptive event, were expressed by the performance function based on three system states: (a) planned/stable state, (b) disrupted state, and (c) planned/stable recovered state. This approach was also applied by the authors for assessing resilience of the given road network, and then for assessing the resilience of container terminals affected by different disruptive events (Pant et al. 2014). Furthermore, a clear difference between engineering and ecological interpretations of concepts of resilience and vulnerability and linked them to connectivity/accessibility in transport networks as well was carried (Reggiani et al. 2015). An additional contribution was the reviewing research on transport system resilience and vulnerability of transport systems indicating that it become a mature field with a well-developed methodology and respectable quantity of research findings with substantive potential for practical applications (Mattsson and Jenelius 2015). In addition, a methodology for evaluating the effectiveness of an increase in capacity on alternative links of public transport networks at the strategic level aiming at mitigating the impacts of disruptive events was developed and applied to Stockholm public transport network. The aim was to indicate how such model could support the network design (Cats and Jenelius 2015). As well, a new approach for estimating the resilience based on a mean-reverting stochastic model studying the diffusive effects of shocks and speed of recovery of the affected system was developed and applied to the case of the London Underground (D’Lima and Medda 2015).
- *Optimization models* dealt with different aspects of optimization of resilience. For example, the mathematical (stochastic-integer) model for evaluation and optimization, i.e., maximization, of the resilience of an airport runway and taxiway network was developed (Faturechi et al. 2014). In particular, the model dealt with the time of restoration of the airside capacity after the end of impact of a given disruptive event. Next was the multi-objective three-stage stochastic model to quantify and optimize the travel time resilience in the road network disrupted according to the specified scenario(s) (Faturechi and Miller-Hooks 2014). In addition, the resilience of a metropolitan public transport network was analyzed by developing a two-stage stochastic programming model. The aim was to deal with resilience as the proportion of satisfied demand by the affected network just after the end of impact of disruptive event (Jin et al. 2014). Specifically, the mathematical model for evaluation of the critical rail infrastructure in order to maximize the resilience of given rail network was developed. The criticality was measured by additional delays imposed by the disrupted component-node or link of the network (Khaled et al. 2015). In addition, Vugrin et al. (2014) proposed the two-level multi-objective optimization model for recovery of a disrupted transport network. The first level dealt with solving the network flows and the second level with the optimal sequence of the recovery actions;
- *Simulation models* have been developed to measure the indicators of resilience and model the impacts of disruptive events through increased travel time and reduction of capacity of the railway transport system (Adjetey-Bahun et al. 2014); and
- *Fuzzy logic models* have dealt with assessing the resilience of critical infrastructure including transport infrastructure where redundancy and adaptability have been considered as the primary components of resilience (Muller 2012).

With the exception of the work of Jianhuai et al. (2013), the above-mentioned research has not explicitly dealt with the resilience of rail passenger networks affected by particularly large-scale disruptive events. Therefore, the added value of the presented research is expected to be as follows:

- Filling in the gap in the existing body of the above-mentioned research respecting explicitly both types of approach and the specificity of the considered case. One of the main reasons is the relative rarity of large-scale disruptions of HSR networks relative to the scope and scale of their operations compared to their counterparts, for example, conventional rail, road, and air passenger transport networks;
- Considering simultaneously different types of inherently dependent performances of the given rail networks and/or their components dynamically, i.e., over time before, during, and after the impact of a given large-scale disruptive event(s);
- Developing sufficiently generic indicators of particular/selected performances by existing, slightly modified, and/or innovative but essentially generic analytical models to be used as the figures-of-merit for assessing resilience of the rail passenger networks of any of the above-mentioned topologies affected by different large-scale disruptive events; and
- Enabling assessment of the resilience of rail passenger networks and their components for different “what-if” scenarios of impacts of disruptive events respecting their diversity, intensity, duration, and scale and scope of impacts.

Consequently, modeling the resilience of rail passenger networks affected by large-scale disruptive event(s) presented in this paper partially follows modeling of the resilience of an air transport network affected by a given (large-scale) disruptive event (Janić 2015), and the generic quantitative metrics to assessing the systems’ resilience depending on time (Henry and Ramirez-Marquez 2012).

### Objectives and assumptions

The objectives of this paper are to model the dynamic, i.e., time-dependent, resilience of a given rail passenger network affected by a given disruptive event(s) at the three above-mentioned levels. The modeling has resulted in developing aa methodology based on the following assumptions:

- The rail passenger network, either conventional or HSR, consists of infrastructure—rail lines and stations along them—where any pair of stations along a given line can be the O–D (Origin–Destination) of user/passenger flows and consequent transport services, thus defining the particular routes; this implies that several routes can simultaneously exist along the same line(s);
- Time, location, intensity, duration, and consequences of impact of a given large-scale disruptive event on the performances of a given rail network are either known from the past case(s) or can also be set up (for example by simulation) to recently occurred and/or the future (hypothetical) case(s), the latter usually according to the “what-if” scenario approach;
- The direct impact of a disruptive event causing gradual or immediate deterioration of the planned infrastructural, operational, economic, and social-economic performances of a given rail network has been exclusively considered; the indirect impacts on the surrounding economic activities/business of the affected stakeholders/parties have not been considered;
- The indicators of particular performances as the figures-of-merit used for assessing resilience relate to the direct costs of damages imposed on particular main stakeholders/actors involved—the rail operator(s), users/passengers, and consequently society;
- Despite being inherently mutually dependent, particular performances and their indicators and considered as independent on each other; and
- After the end of impact of disruptive event, recovery of the affected performances can start and last for some time, i.e., during the recovery period.

### Structure of the methodology

The proposed methodology consists of two sets of models: (1) an analytical model for assessing dynamic resilience of the affected rail passenger network and/or its particular components; and (2) analytical models for estimating indicators of the infrastructural, operational, economic, and social-economic performances as the figures-of-merit for assessing resilience of the affected network under given conditions. Such structuring is made due to the following reasons: (1) the first model of generic structure has been already used in different applications; in the given context it has been just modified to serve the purpose; and (2) the second set contains innovative models originally developed for estimating indicators of particular network’s performances to be used as the figures-of-merit for assessing its resilience means by the above-mentioned generic model.

#### Model of resilience

A generic model of resilience of a given rail passenger network and its components affected by the impact of a given disruptive event has been developed for the following scenario:

1. Resilience can be estimated with respect to the indicators of selected performances as the figures-of-merit;
2. The selected performances and their indicators as the figures-of-merit are in the planned state before the impact of a given disruptive event(s);
3. The impact of a given disruptive event lasting for some time continuously deteriorates the planned state of particular performances and their indicators at a certain rate, thus bringing them to the affected/deteriorated state;
4. After the end of impact of the disruptive event, the recovery of the particular previously affected performances and their indicators can start immediately or after some (preparation) time; this recovery can take place at a certain rate by applying some of the above-mentioned mitigating strategies, thus influencing the corresponding resilience and the recovery time; and
5. At the end of the recovery period, the previously affected indicators of performances and corresponding resilience recover to the full planned state like before the impact of the disruptive event.

Let the line (*i*) of a given conventional or HSR network contain (*M*_*i*_) routes each defined by a couple of end stations as O–Ds (Origin and Destination) of user/passenger flows and corresponding transport services. (*j *= 1, 2,…, *M*_*i*_). The static resilience of the line (*i*) with respect to the indicator (*l*) as the figure-of merit of a given performance (*k*) can be estimated as the ratio between its actually realized and planned value during a given period of time, as follows (Chen and Miller-Hooks 2012; Janić 2015):

1 $$ r_{i/kl} (\tau ) = \sum\limits_{j = 1}^{{M_{i} }} {\left( {\frac{{FOM_{i/j/kl}^{*} (\tau )}}{{FOM_{i/j/kl} (\tau )}}} \right)} {\kern 1pt} {\kern 1pt} {\kern 1pt} {\kern 1pt} {\kern 1pt} \le 1.0{\kern 1pt} {\kern 1pt} {\kern 1pt} or{\kern 1pt} {\kern 1pt} {\kern 1pt} 100\% $$

where

$$FOM_{{i}/{j}/{kl}}^{*} (\tau ) $$
is the actually realized indicator (*l*) of performance (*k*) on the route (*j*) of line (*i*) operating under disruptive conditions during time (*τ*);
*FOM*_*i/j/kl*_(*τ*): is the planned indicator (*l*) of performance (*k*) on the route (*j*) of line (*i*) operating under regular conditions during time (*τ*);
*k*: is type of performance of the route (*j*) of line (*i*) to which its resilience during time (*τ*) is estimated (*k *= 1, 2, …, *K*_*j*_);
*K*_*j*_: is the number of performances considered while dealing with the resilience of route (*j*);
*l*: is the indicator of performance (*k*) of the route (*j*) of line (*i*) respecting to which its resilience is considered (*l *= 1, 2, …, *L*_*j/k*_);
*L*_*j/k*_: is the number of indicators of performance (*k*) considered while dealing with the resilience of route (*j*); and
*τ*: is the time period;

From Eq. 1, the static resilience of a given rail network consisting of (*N*) lines with respect to the selected indicator (*l*) of performance (*k*) can be estimated as follows:

2 $$ R_{kl} (N,\tau ) = \sum\limits_{i = 1}^{N} {r_{i/kl} (\tau )} = \sum\limits_{i = 1}^{N} {\sum\limits_{j = 1}^{{M_{i} }} {\left( {\frac{{FOM_{i/j/kl}^{*} (\tau )}}{{FOM_{i/j/kl} (\tau )}}} \right)} } {\kern 1pt} {\kern 1pt} {\kern 1pt} \le 1.0{\kern 1pt} {\kern 1pt} {\kern 1pt} \hbox{or} {\kern 1pt} {\kern 1pt} {\kern 1pt} {\kern 1pt} 100\% $$

where all symbols are analogous to those in the previous Eq. 1.

As can be seen, Eq. 2 indicates that the resilience of a given rail network with respect to the selected indicator of a given performance increases in line with its actual realization. When the actually realized indicator of performance is equal to the planned one, the corresponding resilience will be equal to 1 (or 100%).

**Fig. 2:**
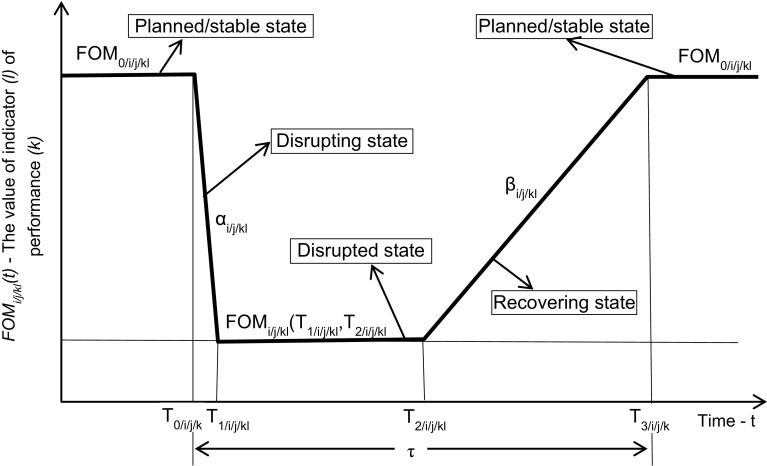
Simplified scheme of the indicator (*l*) of performance (*k*) as the figure-of-merit of the route (*j*) of line (*i*) of a given rail network over time, i.e., just before, during, and after the impact of a given disruptive event (*T*_*3/i/j/kl*_ − *T*_*0/i/j/kl*_=* τ*)

The dynamic resilience depending on the changing of indicator (*l*) of performance (*k*) of the affected route (*j*) of line (*i*) of a given rail network is developed based on the simplified scheme shown on Fig. 2.

Then, the dynamic resilience of the route (*j*) of the line (*i*) respecting the indicator (*l*) of performance (*k*) depending on time (*t*) can be estimated as follows (Henry and Ramirez-Marquez 2012; Janić 2015).

3 $$ \begin{aligned} r_{i/j/kl} (t) = \left[ {\begin{array}{*{20}l} {1,\begin{array}{*{20}c} \quad {for} & {t < T_{0/i/j/kl} } \\ \end{array} } \hfill \\ {1 - \frac{{\alpha_{i/j/kl} \cdot t}}{{FOM_{0/i/j/kl} }},\begin{array}{*{20}c} \quad{for} & {T_{0/i/j/kl} < t < T_{1/i/j/kl} } \\ \end{array} } \hfill \\ {\frac{{FOM_{i/j/kl} (T_{1/i/j/kl} ,T_{2/i/j/kl} )}}{{FOM_{0/ij/kl} }},\begin{array}{*{20}c} \quad{for} & {T_{1/i/j/kl} < t \le T_{2/i/j/kl} } \\ \end{array} } \hfill \\ {\frac{{\beta_{i/j/kl} \cdot t - FOM_{i/j/kl} (T_{1/i/j/kl} ,T_{2/i/j/kl} )}}{{FOM_{0/i/j/kl} - FOM_{i/j/kl} (T_{1/i/j/kl} ,T_{2/i/j/kl} )}},\begin{array}{*{20}c} \quad{for} & {T_{2/i/j/kl} < t \le T_{3/i/j/kl} } \\ \end{array} } \hfill \\ {1,\begin{array}{*{20}c} \quad {for} & {t > T_{3/i/j/kl} } \\ \end{array} } \hfill \\ \end{array} } \right] \le 1.0{\kern 1pt} {\kern 1pt} {\kern 1pt} {\kern 1pt} {\kern 1pt} {\rm or}{\kern 1pt} {\kern 1pt} {\kern 1pt} 100\% \hfill \\ \hfill \\ \end{aligned} $$

where

*T*_*0/i/j/kl*_, *T*_*1/i/j/kl*_: is the time of starting and ending the impact of a given disruptive event, respectively, on the indicator (*l*) of performance (*k*) of the route (*j*) of line (*i*);
*T*_*2/i/j/kl*_, *T*_*3/i/j/kl*_: is the time of starting and ending recovery, respectively, of the indicator (*l*) of performance (*k*) on the route (*j*) of line (*i*);
*t*: is the time during the observed period;
*FOM*_*0/i/j/kl*_, *FOM*_*i/j/kl*_ (*T*_*1/i/j/kl*_*,T*_*2/i/j/kl*_): is the planned and deteriorated value, respectively, of the indicator (*l*) of performance (*k*) of the route (*j*) of line (*i*) (units); and
*α*_*i/j/kl*_, *β*_*i/j/kl*_: is the rate of deterioration and restoration, respectively, of the indicator (*l*) of performance (*k*) of the route (*j*) of line (*i*) during the impact of disruptive event (units/unit of time)

The value of *FOM*_*i/j/kl*_ (*T*_*1/i/j/kl*_*,T*_*2/i/j/kl*_) can be less than *FOM*_*0/i/j/kl*_ or 0 when the planned value of the indicator (*l*) of performance (*k*) is completely deteriorated. If the rate of deterioration of the indicator (*l*) of performance (*k*) is constant, the time of ending the impact of the given disruptive event can be estimated as: *T*_*1/i/j/kl*_ = *T*_*0/i/j/kl*_ + [*FOM*_*0/i/j/kl*_−* FOM*_*i/j/kl*_(*T*_*1/i/j/kl*_*,T*_*2/i/j/kl*_)]/*α*_*i/j/kl*_. Consequently, the term (*α*_*ij/kl*_*·t*) expresses the value of deteriorated indicator of performance (*k*) by time (*t*) (*t ϵ T*_*0/i/j/kl*_, *T*_*1/i/j/kl*_). If recovery of the indicator (*l*) of performance (*k*) starts just after the end of the impact of the disruptive event, the time (*T*_*2/i/j/kl*_−* T*_*1/i/j/kl*_) will be equal to zero. If the rate of recovering of the indicator (*l*) of performance (*k*) is constant, the time of its full recovery up to the planned value can be estimated as: *T*_*3/i/j/kl*_ = *T*_*2/i/j/kl*_ + [*FOM*_*0/i/j/kl*_−* FOM*_*i/j/kl*_(*T*_*1/i/j/kl*_*,T*_*2/i/j/kl*_)]/*β*_*i/j/kl*_. In this case, the term (*β*_*i/j/kl*_*·t*) represents the value of restored indicator (*l*) of performance (*k*) by time *(t)* (*t ϵ T*_*2/i/j/kl*_*, T*_*3/i/j/kl*_). In addition, Eq. 3 also enables expressing deterioration and recovering of a given indicator of performance by subtracting or adding discrete amounts, respectively, at discrete moments of time.

Based on Eq. 3, the dynamic resilience of a given rail network when the impact of a given disruptive event affects the indicator (*l*) of performance (*k*) of the route (*j*) of the line (*i*) can be estimated as follows:

4 $$ \begin{aligned} R_{\begin{subarray}{l} \\ i/j/kl \end{subarray} } (t) = \left[ {\begin{array}{*{20}l} {1,\begin{array}{*{20}c} \quad{for} & {t < T_{0/i/j/kl} } \\ \end{array} } \hfill \\ {1 - \frac{{\alpha_{i/j/kl} \cdot t}}{{\sum\nolimits_{i = 1}^{N} {\sum\nolimits_{j = 1}^{{M_{i} }} {\sum\nolimits_{k = 1}^{{K_{j} }} {FOM_{0/i/j/kl} } } } }},\begin{array}{*{20}c} \quad{for} & {T_{0/i/j/kl} < t \le T_{1/i/j/kl} } \\ \end{array} } \hfill \\ {\frac{{FOM_{i/j/kl} \left( {T_{1/i/j/kl} ,T_{2/i/j/kl} } \right)}}{{\sum\nolimits_{i = 1}^{N} {\sum\nolimits_{j = 1}^{{M_{i} }} {\sum\nolimits_{k = 1}^{{K_{j} }} {FOM_{0/i/j/kl} } } } }},\begin{array}{*{20}c} \quad{for} & {T_{1/i/j/kl} < t \le T_{2/i/j/kl} } \\ \end{array} } \hfill \\ {\frac{{\beta_{i/j/kl} \cdot t - FOM_{i/j/kl} (T_{1/i/j/kl} ,T_{2/i/j/kl} )}}{{\sum\nolimits_{i = 1}^{N} {\sum\nolimits_{j = 1}^{{M_{i} }} {\sum\nolimits_{k = 1}^{{K_{j} }} {FOM_{0/i/j/kl} } } } - FOM_{i/j/kl} (T_{1/i/j/kl} ,T_{2/i/j/kl} )}},\begin{array}{*{20}c} \quad{for} & {T_{2/i/j/kl} < t \le T_{3/i/j/kl} } \\ \end{array} } \hfill \\ {1,\begin{array}{*{20}c} {t > } & {T_{3/i/j/kl} } \\ \end{array} } \hfill \\ \end{array} } \right] \le 1.0{\kern 1pt} {\kern 1pt} {\kern 1pt} {\kern 1pt} {\kern 1pt} or{\kern 1pt} {\kern 1pt} {\kern 1pt} {\kern 1pt} 100\% \hfill \\ {\kern 1pt} {\kern 1pt} {\kern 1pt} {\kern 1pt} \hfill \\ \end{aligned} $$

where all symbols are analogous to those in Eq. 3.

Equation 4 also enables estimation of the dynamic resilience of the network with respect to the specified indicators of performances when the impact of the disruptive event affects simultaneously several indicators of performances of more than a single component—route/line/station.

#### Models of indicators of performances as the figures-of-merit

The selected indicators of performances as the figures-of-merit for assessing dynamic resilience of the affected rail line and entire network at the three above-mentioned (physical, transport service, and cognitive) levels are given in Table 1.

**Table 1:** Indicators and measures of performances as the figures-of-merit *(FOMs*) for assessing the resilience of a given rail network at three—physical, transport service, and cognitive-level

Type of performance	Indicator—figure-of-merit
(1) Infrastructural (physical level—rail operator(s) perspective)	Length of route(s), line(s), network—(*FOM*_*i/j/1/l*_) (*l *=* 1, 2, 3*)
(2) Operational (transport service level—rail operator(s) perspective) (cognitive level—Rail operator(s) perspective)	Scheduled transport service frequency (*FOM*_*i/j/2/l*_) (*l *=* 1,2,3*) Transport work/capacity (*FOM*_*i/j/3/l*_) (*l *= *1, 2, 3*) Delays of the restored transport services due to operating at reduced speed (*FOM*_*i/j/4/l*_)
(3) Economic (cognitive level—rail Operators perspective) (cognitive level—users/passengers perspective)	The rail operator’s losses of profits from cancelled transport services—line(s), route(s), network—(*FOM*_*i/j/5/l*_) (*l *=* 1, 2, 3*) The rail operator’s losses of profits from users/passengers abandoning restored but delayed transport services (*FOM*_*i/j/6/l*_) (*l *=* 1, 2, 3*) The cost of user/passenger time using restored but delayed transport services (*FOM*_*i/j/7/l*_) (*l *=* 1, 2, 3*)
(4) Social-economic (cognitive level—users/passengers perspective)	Accessibility—The index/ratio of user/passenger trip benefits and corresponding generalized accessibility costs—line(s), route(s), network—(*FOM*_*i/j/8/l*_) (*l *=* 1, 2, 3*)

l = 1 (line(s)); l = 2 (route(s)); l = 3 (network)

Referring to particular performances, deterioration of their planned indicators implies the following: for the infrastructure performances—gradual or immediate closing of the affected lines/routes/stations; for the operational performances—cancellation and/or delaying of the affected transport services; for the economic performances—the costs imposed on the rail operator due to cancelled transport services (the costs of repairing physical damages of the network’s components are not considered) and the costs of user/passenger delays; and for the social-economic performances—the social-economic costs due to deteriorated/lost accessibility and consequently non-realized user/passenger trips (if the above-mentioned strategy ‘input (modal) substitution’ is partially applied, these costs can be mitigated).

In addition, from the perspective of rail operator(s), the physical level relates to the performances of rail infrastructure, supporting facilities and equipment, and rolling stock, i.e., their fast repair after being damaged by the impact of disruptive event(s). The transport service level relates to all aspects of performances of transport services restored after repairing of infrastructure. The cognitive level considered from both rail operator(s) and users/passengers perspective relate to the generally deteriorated economic performances by the impact of disruptive event. In particular, rail operator(s) lose the profits from the cancelled transport services and from those restored but operated at reduced speed and thus delayed. Some users/passengers in addition to losing accessibility of their destinations also react to the restored but delayed transport services by abandoning them.

The analytical models of the above-mentioned indicators are as follows:

1. *Infrastructural performances* Length of route(s), line(s), network:

5 $$ FOM_{i/j/1/1} - l_{i/j} - {\text{ Route}}\,(j)\;{\text{of line}}\;(i) $$

6 $$FOM_{i/j/1/2}-l_{i}-{\text{Line}}\;(i)\;{\text{of the network}} $$

7 $$ FOM_{i/j/1/3} - L\left( {L = \sum\limits_{i = 1}^{N} {l_{i} } } \right) - {\text{ Network}} $$

2. *Operational performances I* Scheduled transport service frequency—route(s), line(s), network [dep/unit of time (h or day)]:

8 $$ FOM_{i/j/2/1} - f_{i/j} (\tau ) - {\text{ Route}}\;(j)\;{\text{of line}}\;(i) $$

9 $$ FOM_{i/j/2/2}-F_{i} (\tau )=\sum\limits_{j = 1}^{{M_{i} }} {f_{i/j} (\tau )}-{\text{Line}}\;(i)\;{\text{of the network}}$$

10 $$ FOM_{i/j/2/3} - F(\tau ) = \sum\limits_{i = 1}^{N} {F_{i} (\tau )} = \sum\limits_{i = 1}^{N} {\sum\limits_{j = 1}^{{M_{i} }} {f_{i/j} (\tau )} }-{\text{Network}} $$

3. *Operational performances II* Transport work/capacity—route(s), line(s), network [p-km/unit of time (h or day)]:

11 $$ FOM_{i/j/3/1} - TW_{i/j} (\tau ) = f_{i/j} (\tau ) \cdot \lambda_{i/j} (\tau ) \cdot s_{i/j} (\tau ) \cdot l_{i/j} - {\text{ Route}}\;(j)\;{\text{of line}}\;(i) $$

12 $$ FOM_{i/j/3/2} -TW_{i} (\tau ) = \sum\limits_{j = 1}^{{M_{i} }} {TW_{i/j} (\tau )} - {\text{ Line}}\;(i)\;{\text{of the network}} $$

13 $$ FOM_{i/j/3/3} - TW(\tau ) = \sum\limits_{i = 1}^{N} {TW_{i} (\tau )} = \sum\limits_{i = 1}^{N} {\sum\limits_{j = 1}^{{M_{i} }} {TW_{i/j} (\tau )} } - {\text{ Network}} $$

4. *Operational performances III* Delays of the recovered transport services due to operating at reduced speed:

14 $$ FOM_{i/j/4/1} - D_{i/j} (\tau ) = f_{ij} (\tau ) \cdot d_{i/j} (\tau ) = f_{i/j} (\tau ) \cdot \sum\limits_{r = 1}^{{R_{i} (\tau )}} {l_{i/j/r} \cdot } \left( {1/v_{i/j}^{r} (\tau ) - 1/v_{i/j} (\tau )} \right) - {\text{Route}}\;(j)\;{\text{of line}}\;(i) $$

15 $$ FOM_{i/j/4/2} - D_{i} (\tau ) = \sum\limits_{j = 1}^{{M_{i} }} {D_{i/j} (\tau )} - {\text{ Line}}\;(i)\;{\text{of the network}} $$

16 $$ FOM_{i/j/4/3} - D(\tau ) = \sum\limits_{i = 1}^{N} {\sum\limits_{j = 1}^{{M_{i} }} {D_{i/j} (\tau )} } - {\text{ Network}} $$

5. *Economic performances I* The rail operator’s losses of profits from cancelled transport services:

17 $$ FOM_{i/j/5/1} - c_{i/j} (\tau ) = p_{i/j/c} (\tau ) \cdot f_{i/j} (\tau ) \cdot s_{i/j} (\tau ) \cdot \lambda_{i/j} (\tau ) \cdot l_{i/j} \cdot y_{i/j} (\tau ) - {\text{ Route}}\;(j)\;{\text{of line}}\;(i) $$

18 $$ FOM_{i/j/5/2} - C_{i} (\tau ) = \sum\limits_{j = 1}^{{M_{i} }} {c_{i/j} (\tau )} - {\text{ Line}}\;(i)\;{\text{of network}} $$

19 $$ FOM_{i/j/5/3} - C(\tau ) = \sum\limits_{i = 1}^{N} {C_{i} (\tau )} = \sum\limits_{i = 1}^{N} {\sum\limits_{j = 1}^{{M_{i} }} {c_{i/j} (\tau )} } - {\text{ Network}} $$

6. *Economic performances II* The rail operator’s losses of profits from users/passengers abandoning restored but delayed transport services:

20 $$ FOM_{i/j/6/1} - cd1_{i/j} (\tau ) = f_{i/j} (\tau ) \cdot s_{i/j} (\tau ) \cdot \Delta \lambda_{i/j}^{ - } \left[ {d_{i/j} (\tau )} \right] \cdot l_{i/j} (\tau ) \cdot y_{i/j} - {\text{Route}}\;(j)\;{\text{of line}}\;(i) $$

21 $$ FOM_{i/j/6/2} - CD1_{i} (\tau ) = \sum\limits_{j = 1}^{{M_{i} }} {cd1_{i/j} (\tau )} - {\text{ Line}}\;(i)\;{\text{of the network}} $$

22 $$ FOM_{i/j/6/3} - CD1(\tau ) = \sum\limits_{i = 1}^{N} {CD1_{i} (\tau )} = \sum\limits_{i = 1}^{N} {\sum\limits_{j = 1}^{{M_{i} }} {cd1_{i/j} (\tau )} } - {\text{ Network}} $$

7. *Economic performances III* The cost of user/passenger time using the delayed transport services:

23 $$ FOM_{i/j/7/1} - cd2_{i/j} (\tau ) = f_{i/j} (\tau ) \cdot s_{i/j} (\tau ) \cdot \left( {\lambda_{i/j} (\tau ) - \Delta \lambda_{i/j}^{ - } [d_{i/j} (\tau )]} \right) \cdot d_{i/j} (\tau ) \cdot \theta_{i/j} - {\text{Route}}\;(j)\;{\text{of line}}\;(i) $$

24 $$ FOM_{i/j7/2/2} - CD2_{i} (\tau ) = \sum\limits_{j = 1}^{{M_{i} }} {cd2_{i/j} (\tau )} - {\text{ Line}}\;(i)\;{\text{of the network}} $$

25 $$ FOM_{i/j/7/3} - CD2(\tau ) = \sum\limits_{i = 1}^{N} {CD2_{i} (\tau ) = } \sum\limits_{i = 1}^{N} {\sum\limits_{j = 1}^{{M_{i} }} {cd2_{i/j} } } (\tau ) - {\text{ Network}} $$

8. *Social-economic performances* Accessibility—The index/ratio of user/passenger trip benefits and the corresponding generalized accessibility costs:

26 $$ FOM_{i/j/8/1} - A_{i/j} (\tau ) = p_{i/j}^{c} (\tau ) \cdot \left( {\frac{{l_{i/j} \cdot {\kern 1pt} {\kern 1pt} gdp_{i/j} (\tau )}}{{\theta_{i/j} (\tau ) \cdot t_{i/j} + F_{i/j} (l_{ij} )}}} \right) = p_{i/j}^{c} (\tau ) \cdot \left\{ {\frac{{l_{i/j} \cdot {\kern 1pt} {\kern 1pt} gdp_{i/j} (\tau )}}{{\left[ {\theta_{i/j} (\tau ) \cdot \left( {\frac{1}{2} \cdot \frac{\tau }{{f_{i/j} (\tau )}} + \frac{{l_{i/j} }}{{v_{i/j} (\tau )}}} \right) + F_{i/j} (l_{i/j} )} \right]}}} \right\} - {\text{ Route}}\;(j)\;{\text{of line}}\;(i) $$

27 $$ FOM_{i/j/8/2} - A_{i} (\tau ) = \sum\limits_{j = 1}^{{M_{i} }} {A_{i/j} (\tau )} {\kern 1pt} {\kern 1pt} {\kern 1pt} {\kern 1pt} {\kern 1pt} {\kern 1pt} - {\text{ Line}}\;(i)\;{\text{of the network}} $$

28 $$ FOM_{i/j/8/3} - A(\tau ) = \sum\limits_{i = 1}^{N} {A_{i} (\tau ) = } \sum\limits_{i = 1}^{N} {\sum\limits_{j = 1}^{{M_{i} }} {A_{i/j} (\tau )} } {\kern 1pt} {\kern 1pt} {\kern 1pt} {\kern 1pt} {\kern 1pt} {\kern 1pt} - {\text{ Network}} $$

where

*τ*: is the time of impact and recovery of the affected rail route, line, and/or network (h, days)
*l*_*i/j*_*, l*_*i*_*, L*: is the length of route (*j*) of line (*i*), line (*i*), and of the given network, respectively (km);
*l*_*i/j/r*_: is the length of the (*r*)th segment of the route (*j*) of line (*i*) where recovered transport services operate at reduced speed, respectively (km);
*v*_*i/j*_(*τ*), $$ v_{i/j}^{r} (\tau ) $$
is the planned and reduced operating speed of transport services on the (*r*)th segment of the route (*j*) of line (*i*) during time (*τ*) ((km/h);
*R*_*i*_ (*τ*): is the number of routes (*j*) of line (*i*) where transport services operate at a reduced speed during the time (*τ*) (−);
*f*_*i/j*_(*τ*): is the number of transport service frequencies scheduled on the route (*j*) of line (*i*) during time (*τ*) (dep/h or dep/day);
$$ p_{i/j}^{c} (\tau ) $$
is the proportion of cancelled transport services on the route (*j*) of line (*i*) during time (*τ*) (*p*_*ij/c*_(*τ*)≤* 1.0*) (−);
*s*_*i/j*_(*τ*), *λ*_*i/j*_*(τ)*: is the average seating capacity and average load factor, respectively, of a transport service operating on the route (*j*) of line (*i*) during time (*τ*) (seats/service; -);
*d*_*i/j*_: is the average delay of a transport service operating at a reduced speed on the route (*i*) of line (*j*) during time (*τ*) (min or h/service) (min or h);
$$ \Delta \lambda_{i/j}^{ - } [d_{i/j} (\tau )] $$
is the average decrease of load factor of the delayed transport service for time *d*_*i/j*_(*τ*) on the route (*j*) of line (*i*) during time (*τ*); ($$ \Delta \lambda_{i/j}^{ - } [d_{i/j} (\tau )] $$ ≤* λ*_*i/j*_(*τ*)) (−);
*y*_*i/j*_(*τ*): is the average rail operator’s profits (yield) on the route (*j*) of line (*i*) during time (*τ*) ($US or €/p-km);
*F*_*i/j*_(*l*_*i/j*_): is the average (basic) fare charged to user/passenger traveling on the route (*j*) of line (*i*) ($US/p)
*θ*_*i/j*_(*τ*): is the average *VOT* (Value of Time) of a user/passenger traveling on the route (*j*) of line (*i*) during time (*τ*) ($US or €/p-h); and
*gdp*_*i/j*_(*τ*): is the average socio-economic contribution by a user/passenger trip on the route (*j*) of line (*i*) during time (*τ*) to local/regional/national GDP ($US/p-km)

The other symbols area analogous to those in the previous Eqs.

The above-mentioned performances as the figures-of-merit impact each other. For example, *FOM*_*1*_ immediately causes *FOM*_*5*_; *FOM*_*2*_ cannot exist and sustain without *FOM*_*1*_, and *FOM*_*3*_ without *FOM*_*2*_; *FOM*_*6*_ and *FOM*_*7*_ are caused by *FOM*_*4*_; *FOM*_*8*_ is caused by *FOM*_*1*_.

## An application of the methodology to the affected HSR **(**high speed rail) network

### Input

#### The case

The proposed methodology is applied to assessing dynamic resilience of the Japanese HSR Tohoku line and consequently of the entire HSR Shinkansen network affected by a large-scale disruptive event—the Great East Japan Earthquake on 11 March 2011 (Cole et al. 2017).

*Disruptive event* The above-mentioned earthquake had its epicenter approximately 70 km east of the Oshika Peninsula of Tohoku with the highest recorded intensity at its epicenter of 9.0M_w,_^4^ its hypocenter at an underwater depth of about 30 km, and duration of about 6 min (Kalakan and Sevilgen 2011). The impact caused a huge tsunami, and the Fukushima nuclear plant accident in addition to damage to other infrastructure and properties.

**Fig. 3:**
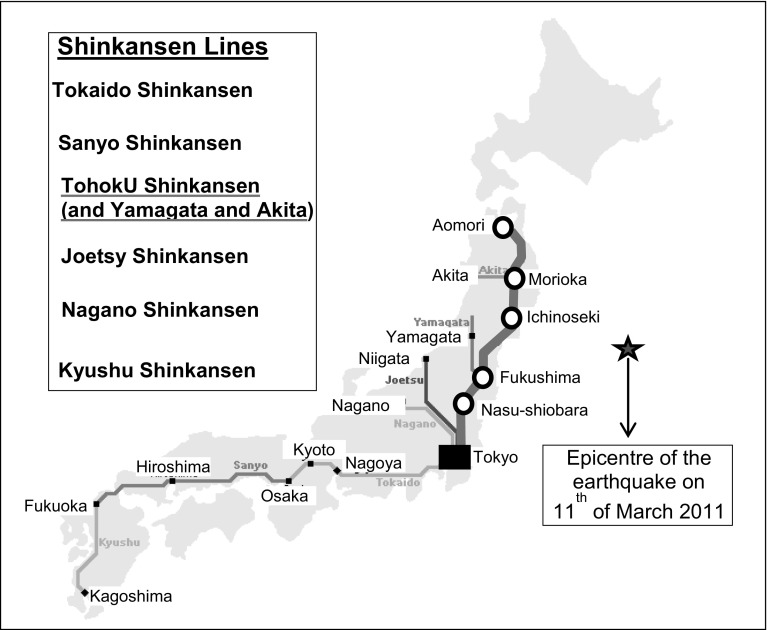
Simplified scheme of the *Japanese HSR Shinkansen network* in the given case.(Reproduced with permission from IHRA 2014; www.japan-guide.com/)

*The affected HSR network* The earthquake also severely impacted the Tohoku line of the Shinkansen HSR network whose simplified schemes are shown on Fig. 3.

As can be seen, the Shinkansen network consists of *N* = 8 main lines (sub-networks) operated by different railway operators/companies. The selected indicators of the planned infrastructural and operational performances of the network and its lines are given in Table 2 (CRJC 2015, JR East 2012, IIHRA 2014).

**Table 2:** Selected indicators of the planned infrastructural and operational performances of the Japanese HSR Shinkansen network in the given example. (Reproduce with permission from IHRA 2014; https://en.wikipedia.org/wiki/Shinkansen/)

Line *(i)*	Length *l*_*i*_ (km)	Service frequency, operating time^a^ *f*_*i*_ (*T*)*, T* (trains/day)	Seating capacity *s*_*i*_ (seats/train)	Transport work/capacity *TW*_*i*_ (*T*) (10^6^ seat-km/day)
1. Tokaido	552.6	323/18	1323	236.142
2. Sanyo	622.3	272/18	1323	223.938
*3. Tohoku* ^*b*^	*713.7*	*235/18*	*510*	*85.537*
4. Yamagata	148.6	36/18	398	2.129
5. Akita	127.3	86/18	723	7.915
6. Joetsu	269.5	96/18	723	118.705
7. Nagano	241.1	114/18	875	29.050
8. Kyushu	256.8	126/18	372	12.037
*Total:*	*2932*	*1288/18*	*–*	*610.454*

^a^Both directions; ^b^Considered affected line

As can be seen, the total length of the considered HSR network is: *L* = 2932 km, with the transport service frequency of: *F*(*T*) = 1341 direct trains (both directions) scheduled during the period of: *T* = 16–18 h/day (IHRA 2014).

*Impact of disruptive event*—*earthquake* Thanks to implementation of the various earthquake-proofing measures before 11/03/2011 based on prior experience of disasters and accidents, none of the 27 HSR trains operating on the Tohoku line directly exposed to the impact of the quake was derailed except for one with no passengers.^5^ In addition, there were no fatalities of users/passengers and crews indicating the robustness of the affected line/network respecting this performance mainly thanks to application of the above-mentioned risk management strategy. Immediately after the end of the quake, the JR East Railway operator applied the above-mentioned ‘conservation’ strategy by suspending all operations of both Shinkansen and local trains along the entire line. On the day after, an investigation of the scale and scope of damages was undertaken. The primary visual inspections carried out by walking along the line detected about 1200 damaged areas along and beneath the elevated railroads. Most frequent and widespread were damages of the electric cable poles and the cables themselves. The concrete structures such as supporting pillars of the elevated rails were with bends or cracks, but none fell or collapsed mainly thanks to the preventive earthquake-resistant reinforcement work carried out prior to the event. Thus, recovering the line’s performances and corresponding resilience by applying the above-mentioned strategies could begin (Jun 2012; Shimamura and Keyaki 2013). As such, this case could be considered as rather typical since earthquakes are relatively common in Japan, frequently impacting the HSR network at varied scales and scopes (Jun 2012).^6^

#### Indicators of the infrastructural and operational performances of the HSR Shinkansen Tohoku line under regular conditions

Some relevant planned indicators of the infrastructural and operational performances of the HSR Shinkansen Tohoku line (No. 3 in Table 2) as the figures-of-merit (*FOMs*) estimated by Eq. 2–3 are given in Table 3.

**Table 3:** Indicators of the planned infrastructural and operational performances as the figures-of-merit of the Tohoku line (*FOM*_*3/j/1/1*_* ≡ l*_*3/j*_; *FOM*_*3/j/2/1*_* ≡ f*_*3/j/2/1*_; *FOM*_*3/j/3/1*_* ≡ TW*_*3/j/3/2*_). (Reproduce with permission from IHRA 2014; www.jreast.co.jp/; https://en.wikipedia.org/wiki/Thoku_Shinkansen/)

Route *(j)*	Length *l*_*i/j*_ (km)	Capacity *s*_*i/j*_ (seats/train)	Service frequency^a^/operating time *f*_*i/j*_* /*(*T*) (dep/day)/(h)	Transport work/capacity *TW*_*i/j*_*(T)* (10^6^ s-km/day)
Tokyo–Nasu-Shiobara *(j *= *1)*	152.4	532	70/18	5.68
Morioka–Shin-Aomori *(j *= *2)*	178.4	731	74/18	9.65
Morioka–Ichinoseki *(j *= *3)*	90.2	725	78/18	5.10
Nasu-Shiobara–Fukushima *(j *= *4)*	102.7	338	38/18	1.32
Tokyo–Shin-Aomori *(j *= *5)*	713.7	731	28/18	14.61
Total	713.7		288/18	36.36

^a^Both directions

As can be seen, the line contained five main routes (*M*_*i*_ = 5) where the HS trains of different seating capacity (*s*_*3/j*_) and service frequency (*f*_*3/j*_) operated during the period of: *T *= 18 h/day. The rest of the time was devoted to the maintenance works (JR 2012). Consequently, the total daily service frequency on all routes in both directions was: *F*_*3*_(*T*) = 288 departures/day. The total line transport work/capacity was: *TW*_*3*_(*T*) = 36.36 · 10^6^ seat-km/day.

#### Indicators of infrastructural and operational performances of the affected HSR Shinkansen Tohoku line

**Fig. 4:**
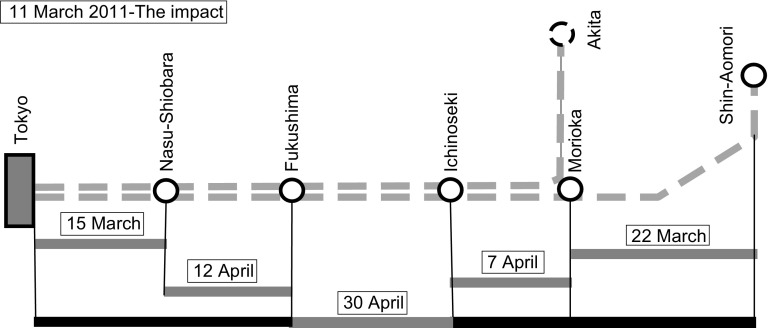
Simplified scheme of the recovery of particular segments/routes of the Tohoku line over time

The impact of the above-mentioned earthquake lasted about: (*T*_*1/3*_ − *T*_*0/3*_)= 6 min. Since all trains on the HSR Shinkansen Tohoku line were stopped and/or suspended during that time, the intensity of impact from Table 3 was estimated to be: *α*_*3/j/1*_ = 713.7/6 = 118.95 km/min, *α*_*3/j/*2_ = 288/6 = 48 transport services/min, and *α*_*3/j/*3_ = 36.36 ·10^6^/6 = 6.06·10^6^ s-km/min. As mentioned above, just after the inspection of damages, resilience strategies focused on recovering the infrastructural, and restoring the operational and economic performances, began to be implemented, as shown in Fig. 4 (Jun 2012; Shimamura and Keyaki 2013).

As can be seen, recovery started from both ends of the line implicitly indicating application of the above-mentioned strategies such as ‘conservation’ first, followed (more or less simultaneously) by ‘management of effectiveness’, ‘import substitution’, ‘logistics refinement’, ‘removing operational impediments’, ‘speeding restoration’ and ‘production recapture’. There is no evidence of the application of the ‘input’ or ‘modal substitution’ strategies (Jun 2012; Shimamura and Keyaki 2013).

In addition, by using Eq. 2–3, the indicators of particular infrastructural and operational performances in terms of the cumulative values of figures-of-merit during application of the recovery/restoring strategies have been estimated and are given in Tables 4 and 5.

**Table 4:** Indicators of infrastructural and operational performances as the figures-of-merit during the recovery/restoring time of the affected Tohoku line—Route length—*FOM*_*3/j/1/1*_* ≡ l*_*3/j/1/1*_; service frequency—*FOM*_*3/j/2/1*_* ≡ f*_*3/j/2/1*_; transport work/capacity—*FOM*_*3/j/3/2*_* ≡ TW*_*3/j/3/2*_. (Reproduce with permission from Jun 2012; Shimamura and Keyaki 2013)

Route *(j)*	Time of restoring *t* (date)	Restoring period *Δ*_*3/j*_ (days)	Cumulative route length/seat capacity *l*_*3/j*_ (km/(seats/train))	Cumulative service frequencies *f*_*3/j*_ (trains/day)	Cumulative transport work/capacity *TW*_*3/j*_ (10^6^ s-km)
	11–03	0	0	0	0
Tokyo–Nasu-shiobara *(j *= *1)*	15–03	4	152.4/532	70	5.68
Morioka–Shin-Aomori *(j *= *2)*	22–03	11	330.8/731	74	34.82
Morioka—Ichinoseki *(j *= *3)*	07–04	27	421.0/725	78	20.43
Nasu-Shiobara—Fukushima *(j *= *4)*	12–04	32	523.7/338	38	21.75
Tokyo—Shin-Aomori *(j *= *5)*	30–04	50	713.7/731	28	36.36

**Table 5:** Indicator of the operational performances as the figures-of-merit due to operating at reduced speed after recovery of the affected Tohoku line—delays—*FOM*_*3/j/4/2*_ ≡ *D*_*3/j/4/2*_. (Reproduce with permission from Jun 2012)

Route *(j)*	Length/restoring time $l_{3/j}/\Delta t_{3/j}$ (km)/(day)	Frequency/affected distance *l*_*3/j/r*_ (dep/day)/(km)	Speed regular/reduced *v*_*3/j/*_$v_{3/j}^{r} (\tau )$ (km/h)	Delay *d*_*3/j*_ (min/dep)	Delays during the prolonger time *Δt*_*3/j*_ *· f*_*3/j*_ *· d*_*3/j*_ (min/τ)	Cumulative delays $D_{3/j}^{*}/ D_{3/j}$^b^ (h/τ)
Morioka–Ichinoseki *(j *= *3)*	90.2/27	78/90.2	135/131	1.2	(119–27)·78·1.2	47.84/143.52
Nasu-Shiobara–Fukushima *(j *= *4)*	102.7/32	38/102.7	146/131	4.8	(119–32)·38·4.8	79.42/378.99
Tokyo–Shin-Aomori*(j *=* 5)*	713.7/50	28/344	211/163	28.8	(119–50)·28·28.8	94.97/1119.73
Fukushima–Ichinoseki^a^ *(j *=* 5′)*	713.7/119	28/151	211/163	12.6	(120–119)·28·12.6	95.38/12.70

^a^Sub-route of the route (*j* = 5)

^b^*D*_3/j_^*^ cumulative delays under regular operating conditions (average unit delay under regular operating conditions—0.6 min/dep) (IHRA 2014)

As can be seen, it has been assumed that (*T*_*2/3/j/k*_ − *T*_*1/3/j/k*_)= 0, i.e., the impact was almost instant (*M*_*i*_ = 5; *K*_*j*_ = 3). In addition, complete line recovery was achieved in 50 days. Each applied strategy resulted in recovering the infrastructure and then restoring the full planned (pre-impact) daily service frequencies and corresponding transport work/capacities, all during the regular daily operating time (18 h). However, after opening the full route length (Tokyo-Shin-Aomori), the HS trains had to operate at reduced speeds over a prolonged period of time in order to maintain the required level of safety, which caused delays as given in Table 5.

As can be seen, as intuitively expected, delays due to the speed restrictions were much higher than that under regular operating conditions and took place until the lifting of the restrictions on the 119th/120th day after the impact of the disruptive event (8/9 July 2011).

#### Indicators of the economic performances of the affected HSR Shinkansen Tohoku line

The first of three indicators of the economic performances of the affected HSR Shinkansen Tohoku line relates to the costs in terms of losses of the HSR operator’s profits from the cancelled transport services during the impact of the disruptive event and the recovery time. Based on Eq. 5, Table 6 gives an estimation of these losses.

**Table 6:** Indicator of the economic performances as the figures-of-merit of the affected Tohoku line—losses of the HSR operator’s profits from the cancelled transport services—*FOM*_*3/j/5/2*_* ≡ C*_*3/2/5/2*_*(t)*)

Route (*j*)	Restoring period^a^ (*T*_*3/3/j *_− *T*_*2/3/j*_) (days)	Loss^b^ *c*_*3/j*_ (10^6^ $US/day)	Time of loss (*c*_*3/j/4*_) *Δt*_*3/j*_ (days)	Profits/losses during the sub-period *c*_*3/j*_ *·Δt*_*3/j*_ (10^6^ $US)	Cumulative profits/losses^c^ *P*_*3/j*_*(τ*)^*^/*C*_*3/j*_*(τ*) (10^6^ $US)
	0	0	0	0	0
Tokyo–Nasu-Shiobara (*j *= *1*)	4	0.174	4	0.696/0.695	0.696/0.696
Morioka–Shin-Aomori (*j *= 2*)*	11	0.295	7	3.245/2.067	3.941/2.762
Morioka–Ichinoseki (*j *= 3*)*	27	0.156	16	4.455/2.497	8.396/5.259
Nasu-Shiobara–Fukushima (*j *= 4*)*	32	0.404	5	1.280/0.202	9.676/5.461
Tokyo–Shin-Aomori (*j *= 5*)*	50	0.447	18	22.850/8.046	33.526/13.507

^a^Based on Tables 4, 5 and Fig. 4

^b^Based on the average regular load factor of: *λ*_*3/j*_ = 0.90; Average profits-yield: *y*_*3/j*_ = 3.4 ¢$US/p-km; 1$$U.S. = 94 Yen

^c^*P*_*3/j*_*(τ*)^*^—Cumulative operator’s profits under regular operating conditions (JR East 2011)

The second indicator of the economic performances relates to the costs in terms of losses of the HSR operator’s profits from the users-passengers giving up from the restored but delayed transport services due to operating at reduced speed after the full restoring of the line’s transport work/capacity. These costs were estimated by Eq. 7 and are given in Table 7.

**Table 7:** Indicator of the economic performances as the figures-of-merit of the affected Tohoku line—losses of the HSR operator’s profits from the users/passengers abandoning delayed (restored) transport services—*FOM*_*3/j/6/2*_ ≡ *CD1*_*3/j/6/2*_*(t)*)

Route *(j)*	Length/operating time $$ l_{3/j}/\Delta t_{3/j} $$ (km)/(days)	Affected distance *l*_*3/j/r*_ (km)	Speed regular/ reduced *v*_*3/j/*_ $$ v_{3/j}^{r} (\tau ) $$ (km/h)	Frequency/capacity/load factor *f*_*3/j*_* /s*_*3/j*_/*/* $$ \Delta \lambda_{3j}^{ - } $$ (dep/day)/(seats/dep)/(loss)^b^	Unit profits (yield) *y*_*3/j*_ (¢/p-km)^c^	Cumulative profits/losses $$ PD_{3/j}^{*}/ CD1_{3/j} $$ (10^3^$US/τ)
Morioka–Ichinoseki *(j *= *3)*	90.2/(119–27	90.2	135/131	78/725/0.296	3.4	14.367/4.722
Nasu-Shiobara–Fukushima *(j *= *4)*	102.7/(119–3232	102.7	146/131	38/338/0.296	3.4	17.872/5.877
Tokyo–Shin-Aomori *(j *=* 5)*	713.7/(119–50)	344	211/163	28/731/0.135	3.4	32.738/8.107
Fukushima–Ichinoseki^a^ *(j *=* 5’)*	713.7/120–119)	151	211/211	28/731/0.068	3.4	33.683/8.178

^a^Sub-route of the route (*j* = 5

^b^Both directions; based on the average regular load factor of: *λ*_*3/j*_ = 0.90 and the elasticity of demand: − 2.545 for distances ≤ 500 km, and − 0.900 for distances > 500 km (Fu et al. 2014)

^c^Based on: 1$US = 94Yen; *PD*_3/j_^*^—Profits under regular operations (average unit delay: 0.4 min/dep) (IHRA 2014; JR East 2011)

The last indicator of economic performances relates to the costs of the time of users/passengers who used the restored transport services of the recovered HSR Shinkansen Tohoku line despite the delays. These costs were estimated by Eq. 8 and are given in Table 8.

**Table 8:** Indicator of the economic performances as the figures-of-merit of the affected Tohoku line—costs of user/passenger time using the restored but delayed transport services (*FOM*_*3/j/7/2*_ ≡ *CD2*_*3/j*_)

Route (*j*)	Length/time of recovery *l*_*3/j*_* /Δt*_*3/j*_ (km/days)	Length of affected segment *l*_*3/j*_ (km)	Speed *v*_*3/j/*_ $$ v_{3/j}^{r} (\tau ) $$ (km/h)	Delay *d*_*3/j*_ (min/dep)	Frequency/capacity/load factor *f*_*3/j*_* /s*_*/3/j*_/*/λ*_*3/j*_ (dep/day)/(seats/dep)/ (loss)^b^	Value of time (VOT) *θ*_*3/j*_ ($/h)^c^	Cumulative cost of pass. time $$ CDR_{3/j}^{*}/ CD_{3/j} $$ (10^6^$US/τ)
Morioka–Ichinoseki (*j *= 3)	90.2/(119–27)	90.2	135/131	1.2	78/725/0.604	19	0.398/1.194
Nasu-Shiobara–Fukushima (*j *= 4)	102.7/(119–32)	102.7	146/131	4.8	38/338/0.604	19	0.487/2.258
Tokyo–Shin-Aomori (*j *= 5)	713.7/(119–50)	344	211/163	28.8	28/731/0.765	19	0.6235/12.112
Fukushima–Ichinoseki^a^ (*j* = 5^*’*^*)*	713.7/120–119)	151	211/211	27.0	28/731/0.765	19	0.626/12.245

^a^Sub-route of the route: *j* = 5

^b^Both directions; based on the average regular load factor of: *λ* = 0.90, and elasticity of demand of: − 2.545 for distances ≤ 500 km, and − 0.9 for distances > 500 km (Fu et al. 2014)

^c^The number of working hours: 136.2 h/month; 1$US = 94Yen; *CDR*_3/j_^*^—cost of passenger time under regular operations (average delay 0.4 min/dep) (IHRA 2014; JR East 2011)

As can be seen, the cumulative costs of passenger time while using restored but delayed transport services during the period of 92 days (11/04–08/07/2011) reached about 12.245 million $US (Jun 2012).

#### Indicator of the social-economic performances of the affected HSR Shinkansen Tohoku line

The indicator of the social-economic performances of the affected HSR Tohoku line relates to the ratio between the benefits and costs of accessibility as an index during the line’s recovery time of: *τ* = 50 days. This ratio/index was estimated by Eq. 9 and is given in Table 9.

**Table 9:** Indicator of the social-economic performances as the figures-of-merit of the affected Tohoku line (accessibility—*FOM*_*3/j/8/1*_≡ *A*_*3/j*_). (Reproduced with permission from EJRC 2016; SB 2014; https://www.jreast.co.jp/e/charge/index.html; http://www.shinkansen.co.jp/jikoku_hyo/en/touhoku/tuh_shinaomori.htm)

Route (*ij*)	Length/Restoring time *l*_*3/j/ *_*/t*^a^ (km/days)	Total travel time *t*_*3/j*_^b^ (h)	Basic price/fare *F*_*3/j*_(*l*_*3/j*_)^c^ ($US/p)	Restored route accessibility index (*A*_*3/j*_+* A*_*j/3*_)^d^ (−)	Restored route accessibility index over time *Δt*_*3/j*_ *·*(*A*_*3/j*_+* A*_*j/3*_)^d^ (−)	Restored cumulative accessibility index $$ \left( {\sum_{j = 1}^{{M_{3} }} {A_{3/j} } + \sum_{i = 1}^{{M_{3} }} {A_{j/3} } } \right) $$(−)
Tokyo–Nasu -shiobara (*j *= *1*)	152.4/4	1.232	26.61	1.121	4.485	4.485
Morioka–Shin-Aomori (*j *= *2*)	178.4/11	1.267	34.27	1.125	12.380	16.685
Ichinoseki–Morioka (*j *=* 3*)	90.2/27	0.857	18.10	0.965	26.071	42.936
Nasu–shiobara–Fukushima *(j *=* 4*)	102.7/32	0.971	20.82	0.963	30.802	73.738
Tokyo–Shin-Aomori (*j *=* 5*)	713.7/50	3.703	109.08	1.458	72.885	146.623

^a^Time (*t ∈ τ; τ* = 50 days)

^b^Based on the schedule delay depending on the transport service frequencies during time: T = 18 h/day and in-vehicle/train travel time depending on the speed-distance relationship (Shinkansen): *v(l) *=* 1.336·ln(l) − 41.565*; *R*^*2*^ = 0.960

^c^Based on the basic fares: *F(l)* =− *7E *− *05·l*^*2*^+* 0.2022·l *+* 0.4264*; *R*^*2*^ = 0.998

^d^Both directions based on: *gdp*_*3/j*_ *(τ)* = 0.184 $US/p-km; *θ*_*3/j*_*(τ)* = 19.0 $US/h (*(i)∈ N*) (IHRA 2014; JR East 2011)

The accessibility index was estimated based on the above-mentioned assumption of not considering the effects of the ‘input’ or ‘modal substitution’ strategy of the cancelled HSR transport services by their, for example, individual car, bus, and/or air transport service counterparts. There was no evidence of the use of the individual car and bus services, while using air transport services would not have been convenient anyway due to the very short routes (except that between Tokyo and Shin-Aomori).

### Analysis of the results

The results from the application of the above-mentioned methodology using the inputs from the given case in Tables 2, 3, 4, 5, 6, 7, 8 and 9 are shown in Figs. 5, 6, 7, 8 and 9 for the affected HSR Shinkansen Tohoku line and in Figs. 10, 11 and 12 for the consequently affected HSR Shinkansen network.

**Fig. 5:**
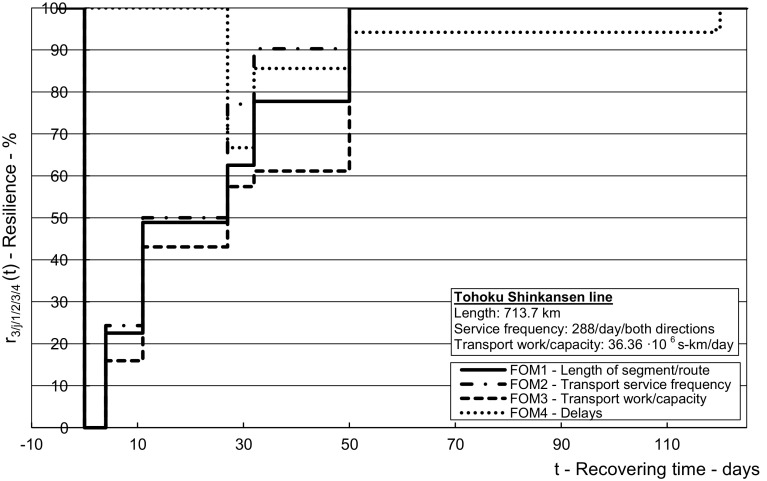
Dynamic resilience respecting the indicators of infrastructural and operational performances during recovering of the affected *Tohoku line*—length of route (*FOM*_*1*_), transport service frequency (*FOM*_*2*_), transport work/capacity (*FOM*_*3*_), delays (*FOM*_*4*_)

**Fig. 6:**
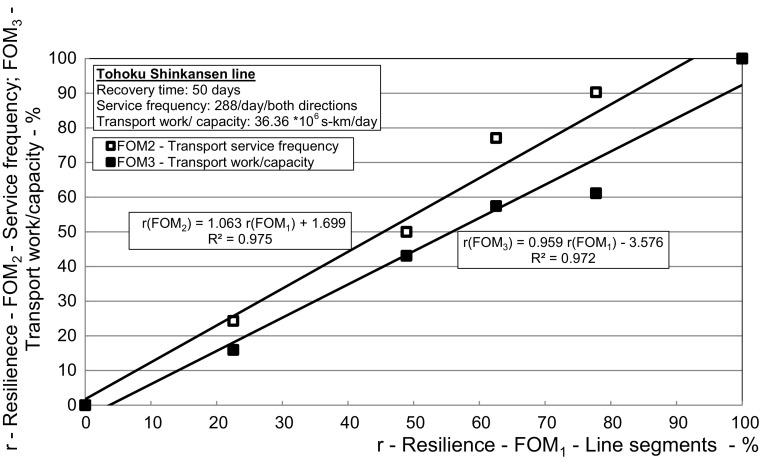
*The Tohoku line*—Relationship between the dynamic resilience respecting the figures-of-merit—transport service frequency (*FOM*_*2*_) and route seating capacity (*FOM*_*3*_) versus the segment/route (*FOM*_*1*_)

**Fig. 7:**
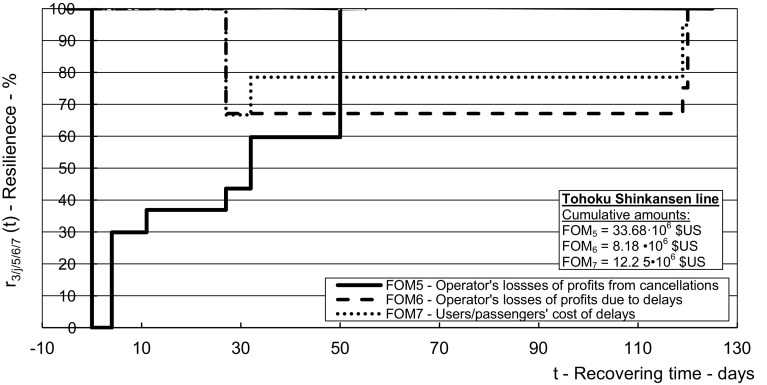
Dynamic resilience respecting the indicators of economic performances during recovery of the affected *Tohoku line*—cost of losses of the operator’s profits from cancelled and delayed transport services (*FOM*_*5*_*, FOM*_*6*_), and the cost of passenger time due to delays (*FOM*_*7*_)

**Fig. 8:**
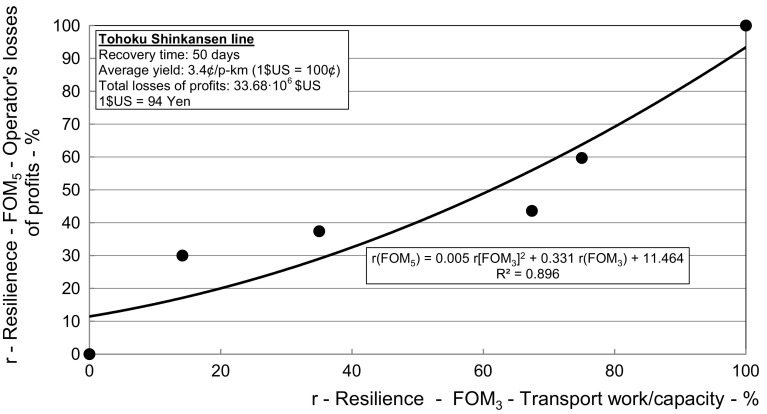
*The Tohoku line*—Relationship between resilience respecting the figures-of-merit—Losses of the HSR operator’s profits from the cancelled transport services (*FOM*_*5*_) versus the transport work/capacity (*FOM*_*3*_)

**Fig. 9:**
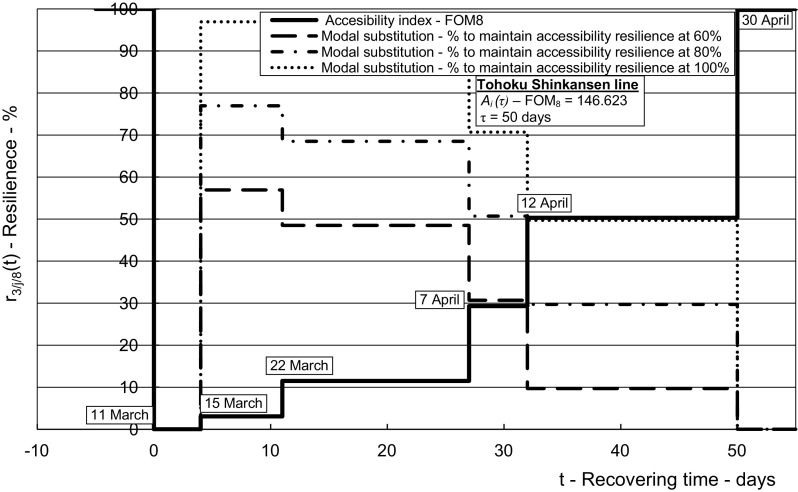
Resilience respecting the indicators of economic performances during recovery of the affected *Tohoku line*—accessibility index (*FOM*_*8*_)

**Fig. 10:**
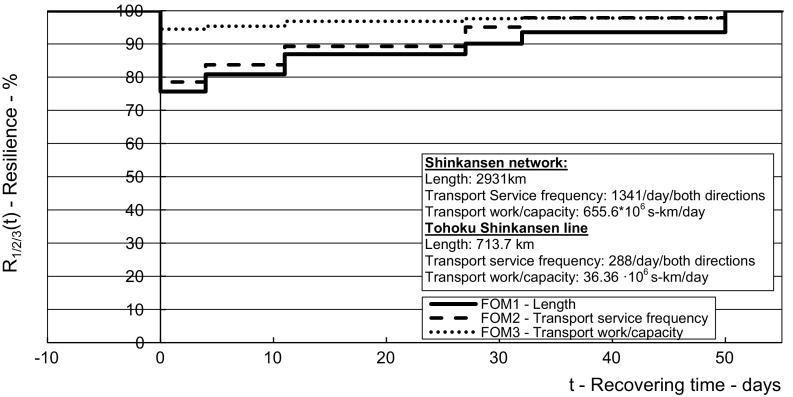
Resilience of the *HSR Shinkansen network* respecting the infrastructural—length of the network (*FOM*_*1*_), and operational performances–transport service frequency (*FOM*_*2*_), and transport work/capacity (*FOM*_*3*_)—influenced by the corresponding *FOMs* of resilience of the affected *Tohoku line*

**Fig. 11:**
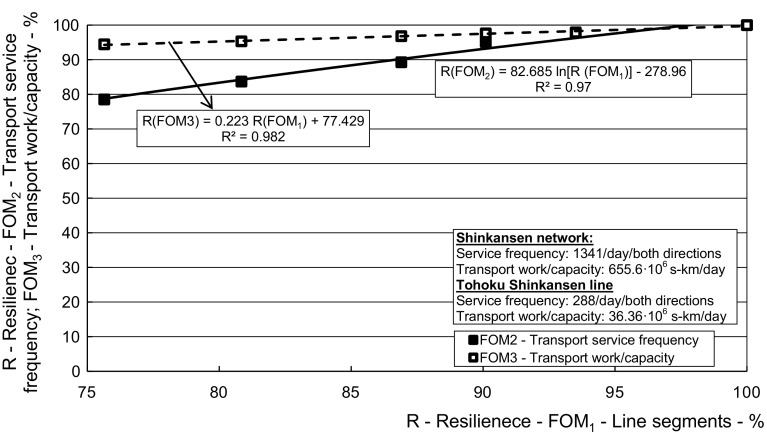
*The HSR Shinkansen network*—relationship between the resilience in terms of the transport work/capacity (*FOM*_*3*_), transport service frequency (*FOM*_*2*_), and length of line (*FOM*_*1*_) influenced by the corresponding *FOMs* of resilience of the affected Tohoku line

**Fig. 12:**
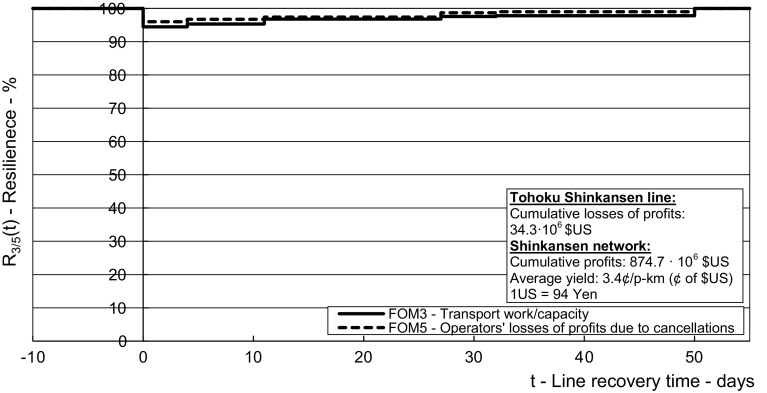
Dynamic resilience of the *The HSR Shinkansen network* respecting the economic—losses of the HSR operator’s profits from cancelled transport services (*FOM*_*5*_) and the operational performances—transport work/capacity (*FOM*_*3*_)—influenced by the corresponding *FOMs* of the affected Tohoku line in the given example

#### The affected Shinkansen Tohoku HSR line

Figure 5 shows the dynamic resilience of the affected HSR Shinkansen Tohoku line depending on the recovered infrastructural and restored operational performances up to their planned/original stable (pre-impact) state.

As can be seen, the resilience with respect to three figures-of-merit (*FOM*_*1*_, *FOM*_*2*_, and *FOM*_*3*_) gradually increased at discrete times, i.e., when the particular segments/routes of the line were recovered and reopened for traffic. At each such segment/route, the resilience of transport service frequency was the highest, followed by that of the length of route/line and the transport work/capacity. This indicates application of the ‘production recapture’ strategy to, immediately after recovering the segment/route, begin and gradually increase the line transport service frequencies, and the corresponding transport work/capacity, although at reduced speeds. In this latest case, the resilience respecting the indicator (*FOM*_*4*_) assumed to be 100% before reopening of the particular segments/routes (due to non-running transport services) started to decrease because of the increase of cumulative delays compared to that under regular operating conditions. It remained compromised by about 6% until removal of the speed restrictions on the 119th/120th day from the impact of the disruptive event. In addition, Fig. 6 shows the dependence of the dynamic resilience in terms of the transport service frequency (*FOM*_*2*_) and the transport work/capacity (*FOM*) on that in terms of the route/line length (*FOM*_*1*_).

As can be seen, both (*FOM*_*2*_) and (*FOM*_*3*_) approximately linearly increase with increasing of (*FOM*_*1*_) thus clearly indicating consistency of application of the above-mentioned strategies—‘management of effectiveness’, ‘import substitution’, ‘speeding restoration’, ‘removing operational impediments’ and ‘production recapture’.

Figure 7 shows the dynamic resilience of the Tohoku line depending on the restored economic performances.

As can be seen, the resilience according to the losses of the HSR operator’s profits from the cancelled transport services recovered in line with the recovering of particular segments/routes and their transport works/capacity. They fully recovered after the whole line and related transport work/capacity recovered (50 days from the impact). In the meantime, some recovered transport services were imposed delays causing users/passengers to abandon them, resulting in losses of the HSR operator’s profits. In addition, the users/passengers who used these services experienced the cost of time which compromised the corresponding resilience by about 20% until the time of removing the speed restrictions (119th/120th day from the impact of the disruptive event).

Figure 8 shows the dependence of the dynamic resilience of the Tohoku line in terms of losses of the HSR operator’s profits from the cancelled transport services (*FOM*_*5*_) and that in terms of the transport work/capacity (*FOM*_*3*_).

As can be seen, the resilience in terms of (*FOM*_*5*_) restored more than proportionally with restoring of the resilience in terms of (*FOM*_*3*_) during the line recovery time.

Figure 9 shows the dynamic resilience of the affected HSR Shinkansen Tohoku line depending on recovering accessibility as the figure-of-merit *FOM*_*8/2*_.

As can be seen, under conditions of not applying the ‘input’ or ‘modal substitution’ strategy, the resilience according to *FOM*_*8*_ (the accessibility index) gradually recovered, as did its infrastructural and operational *FOM*_*1*_, *FOM*_*2*_, *FOM*_*3*_, and *FOM*_*4*_ counterparts. The highest rate was noted toward the end of recovery period, when the longest route recovered, i.e., 50 days from the day of impact. However, if the accessibility resilience of the corridor to be maintained during the recovery of the affected HSR line is specified at the level of say 60, 80, 100% (dotted lines on Fig. 9), assuming that the ‘input’ or ‘modal substitution’ strategy was applied, higher volumes of modal substitution would be needed at the beginning of the line’s recovery period, and would then decrease in line with restoring of the accessibility resilience of the line itself. In addition, if the specified accessibility resilience of the corridor to be maintained was higher, the volume of the modal substitution would need to be more voluminous too, which is as intuitively expected. This indicates that the ‘input’ or ‘modal substitution’ strategy could play an important role in maintaining accessibility resilience in this and other similar cases, and consequently mitigate the corresponding social-economic impacts.

#### The affected Shinkansen network

Figure 10 shows the dynamic resilience of the HSR Shinkansen network depending on the resilience of its affected Tohoku line in terms of the figures-of-merit—length of line (*FOM*_*1*_), transport service frequency (*FOM*_*2*_), and transport work/capacity (*FOM*_*3*_).

As can be seen, the resilience of the HSR Shinkansen Tohoku line in terms of (*FOM*_*1*_) mostly affected the corresponding resilience of the entire network. This was because the Tohoku line was one of the longest in the network. The impact in terms of (*FOM*_*2*_) was less and the impact in terms of (*FOM*_*3*_) the least. In the latest case, this was because although the Tohoku line used to be one of the longest, the other lines had much greater transport work/capacity. Consequently, the resilience of the network in terms of (*FOM*_*3*_) was not compromised by more than about 2–6% during the entire recovery period of the Tohoku line.

Figure 11 shows dependence of the dynamic resilience of the HSR Shinkansen network in terms of the transport service frequency (*FOM*_*2*_) and the transport work/capacity (*FOM*_*3*_) on that in terms of the network length (*FOM*_*1*_) influenced by the corresponding *FOMs* of resilience of the affected Tohoku line.

As can be seen, the resilience of the Shinkansen network in terms of (*FOM*_*2*_) (Transport service frequency) recovered at a higher rate than that in terms of (*FOM*_*3*_) (Transport work/capacity) with recovering of its resilience in terms of (*FOM*_*1*_) (The network length). The main reason was, as mentioned above, that the Tohoku line influenced the network resilience by its length much more than by its transport work/capacity—the latter mainly due to generally lower (*FOM*_*3*_) than that at some other (although shorter) lines.

Figure 12 shows the relationship between the dynamic resilience of the HSR Shinkansen network in terms of losses of the HSR operator’s profits from the cancelled transport services (*FOM*_*4*_) and the transport work/capacity (*FOM*_*3*_) influenced by the corresponding *FOMs* of the affected Tohoku line.

As can be seen, by cancelling all transport services and related transport work/capacity and consequently compromising the corresponding resilience, the resilience in terms of losses of the HSR Shinkansen network operator’s profits from the cancelled transport services was also compromised by about 2–4% during the recovery period of the Tohoku line. After restoring the resilience of the affected (Tohoku) line in terms of its operational performances over the recovery period (50 days), the resilience of the Shinkansen network in terms of the economic performances—losses of the HSR Shinkansen network operators’ profits from the cancelled transport services –also recovered almost proportionally.

## Conclusions

The paper dealt with modeling the resilience of rail passenger transport networks, either conventional or HSR, affected by large-scale disruptive event(s). This modeling resulted in developing a methodology consisting of an analytical model for assessing dynamic resilience with respect to the selected indicators of performances and the models of indicators of these performances used as the figures-of-merit for assessing resilience. Infrastructural, operational, economic, and social-economic performances were considered. The selected indicators of the infrastructural performances were the length of a given conventional or HSR network and its particular routes/lines. The indicators of operational performances were transport service frequencies, the corresponding transport work/capacity, and delays of transport services due to operating at reduced speeds. The indicators of economic performances were the rail operator’s profit losses from the cancelled transport services, the users/passengers abandoning the delayed transport services, and the cost of time of the users/passengers using the delayed transport services. The indicator of the social-economic performances was accessibility in terms of contributions of the user/passenger trips to local/regional/national GDP (Gross Domestic Product).

The methodology was applied to the case of the Japanese HSR (high speed rail) Shinkansen network and its Tohoku line affected by a large-scale disruptive event—the Great East Earthquake, which took place on 11 March 2011. The results from this application indicate that the resilience, depending on all considered indicators of performances—figures-of-merit—of the affected routes of Tohoku line and of the entire Shinkansen network, was affected by the impact of disruptive event. In the case of the Tohoku line, it dropped to zero due to its closing and consequent cancellation of all transport services. During the recovery period, resilience increased gradually in discrete amounts at the times of reopening particular repaired segments/routes and consequent restoration of transport service frequencies and corresponding transport work/capacity. Simultaneously, the resilience depending on the profits/losses of the rail operator’s profits from the cancelled and delayed transport services also gradually recovered in line with recovering of the route/line transport work/seating capacity. At the end of the recovery period, it was fully restored. Resilience in terms of the user/passenger restored accessibility and its contribution to GDP developed in a similar manner.

In addition, through the gradual recovery of the affected line, the resilience of entire HSR Shinkansen network also gradually recovered respecting the particular indicators of performances.

The above-mentioned application indicates the convenience of the proposed methodology to be continued and applied in further research as follows:

1. Assessment and comparison of the resilience of different, both conventional and HSR, networks and their particular components under both actual and hypothetical conditions of occurrence of disruptive events and their impacts, the latter according to the “what-if” scenario approach;
2. Simultaneously and/or individually considering independently and/or depending on each other different types of performances of the affected rail networks and their components over time, i.e., dynamically;
3. Developing additional more detailed case-specific models of indicators and measures of particular performances to be used as the figures-of-merit in existing and/or slightly modified models of resilience of technical systems; and
4. Contributing to partially filling in the missing gap in the existing research.

## Footnotes

1 The conventional rail network is characterized with the infrastructure accommodating in many cases both passenger and freight transport services (trains) carried out at the maximum operation speed of up to 160 km/h. The largest parts of many HSR networks worldwide are dedicated to the passenger transport services carried out at the speed between 200  and 400 km/h (https://en.wikipedia.org/wiki/Passenger_rail_terminology#Conventional_rail/).

2 For example, heavy snowfall in the Calais area (France) in the evening of 18 December 2009 caused the closure of Eurotunnel for 4 days and thus directly affected the HSR traffic and transport between London, Paris, and Brussels. In particular, over the first night of the heavy snowfall five trains failed inside the Channel tunnel (Jaroszweski et al. 2014).

3 As will be seen, an example of the integrated prevention and protection strategy is the earthquake warning system at the Japanese HSR Shinkansen network (Shimamura and Keyaki 2013).

4 The *M*_*w*_ (Moment magnitude scale) is used by seismologists to measure the size of earthquake in terms of the energy released (Utsu 2002).

5 The JR East Railway company implemented the Earthquake Early Detection System with seismometers on 97 locations along its network lines and along the coast to detect preliminary earthquake tremors known to come with a forewarning preliminary tremor and then with a main tremor causing huge quakes. When a seismometer detects a preliminary tremor and judges that the main tremor is to be forceful, it sends an alarm to a substation to cut the power supply to the potentially impacted rail line(s). This causes the HS trains to activate their emergency brakes and stop. In the given case, the time between detecting the preliminary tremor and activating the emergency brakes on the Shinkansen trains operated at the speed of about 275 km/h was 3 s. One minute and seven seconds later the impact began, but at that time all trains had already stopped or were close to stopping (Jun 2012; Kazama and Noda 2012; Shimamura and Keyaki 2013).

6 For example, on 14 April 2016, the Shinkansen Kagoshima Route was closed after the impact of the first of two powerful earthquakes. They caused extensive damage to infrastructure including cracks in the elevated support structures at 25, and collapsed sound-insulation walls at 80 locations including the derailment of a HS train. On 18 April, the resilience actions had begun and parts of the line were opened but with reduced transport service frequencies compared to the planned/stable state of operations (Mainichi Japan 2016).
